# Supplementary material for: Designing nanohesives for rapid, universal, and robust hydrogel adhesion
Source: Nat Commun. 2023 Sep 4;14:5378. doi: 10.1038/s41467-023-40753-5 (PMC10477317; doi:10.1038/s41467-023-40753-5)
Supplement: Supplementary file 1 — Supplementary Information [file 41467_2023_40753_MOESM1_ESM.pdf]

Supplementary Information

Designing nanohesives for rapid, universal and robust hydrogel adhesion

Zhao Pan[1,2†], Qi-Qi Fu[3†], Mo-Han Wang[4†], Huai-Ling Gao[1], Liang Dong[1,2], Pu Zhou[4], Dong-Dong Cheng[4], Ying Chen[3], Duo-Hong Zou[4], Jia-Cai He[4], Xue Feng[5]*, Shu-Hong Yu[1]*

[†]These authors contributed equally to this work.

*Corresponding author: Xue Feng (fengxue@tsinghua.edu.cn), Shu-Hong Yu (shyu@ustc.edu.cn)

This file includes:

Supplementary Table 1

Supplementary Figure S1 to S14

Supplementary References

Other Supplementary Materials for this manuscript include the following:

Supplementary Movie 1 to 10

Supplementary Table

Supplementary Table 1 The adhesive performance of ANP glue in adhering PDMA hydrogels is compared with previously reported nanoparticles-based glues.

**Nanoparticles**	**Lap-shear strength**	**Adhesion energy**	**Elastic modulus**	**Equilibrium swelling degree**	**Adhesion energy calculation**
Silica Nanoparticles[1]		6-10 J/m^2	10 ± 1.0 kPa	41	$G_{adh}=3(F/w)2/(2Eh)$[a]
HAp nanoparticles[2]	2-8 kPa				
PDA-nanoparticles-PVA[3]	1 kPa		45 ± 2 kPa	17	
Colloidal supraballs [4]		10-100 J/m^2	9 kPa<br>18 kPa	3.85<br>2.46	$G_{adh}=3(F/w)2/(2Eh)$[a]
Mesoporous nanoparticles[5]		5-35 J/m^2	10 kPa		$G_{adh}=3(F/w)2/(2Eh)$[a]
Activated silica nanoparticles, this article	~23 kPa	~400 J/m^2	21 ± 8 kPa	620	$G_{adh}=F_p/w$ [b]

Annotation

a, F donate measured adhesive force, w, and h donate the width and thickness of the sample, respectively.

b, Fp donate measured plateau force in the steady-state region of the peeling process, w donate width of the sample.

Supplementary Figures

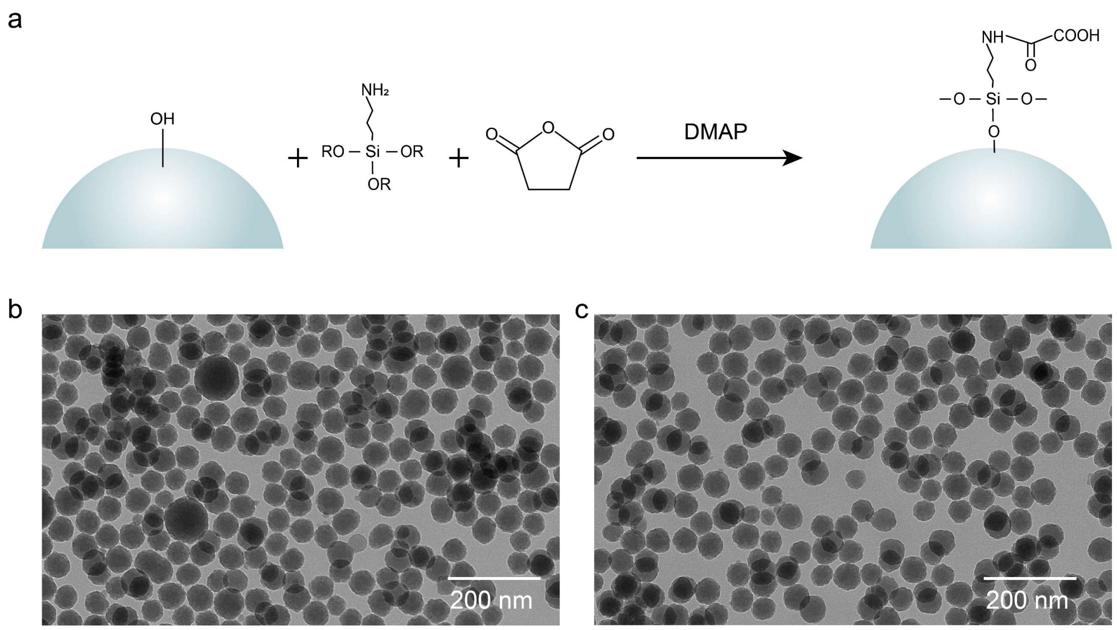

Supplementary Figure 1 a, The silanization modification of silica nanoparticles to activate the surface. b, c, The TEM images of nanoparticles before and after silanization. The experiment was repeated three times independently, and similar results were obtained for TEM observation.

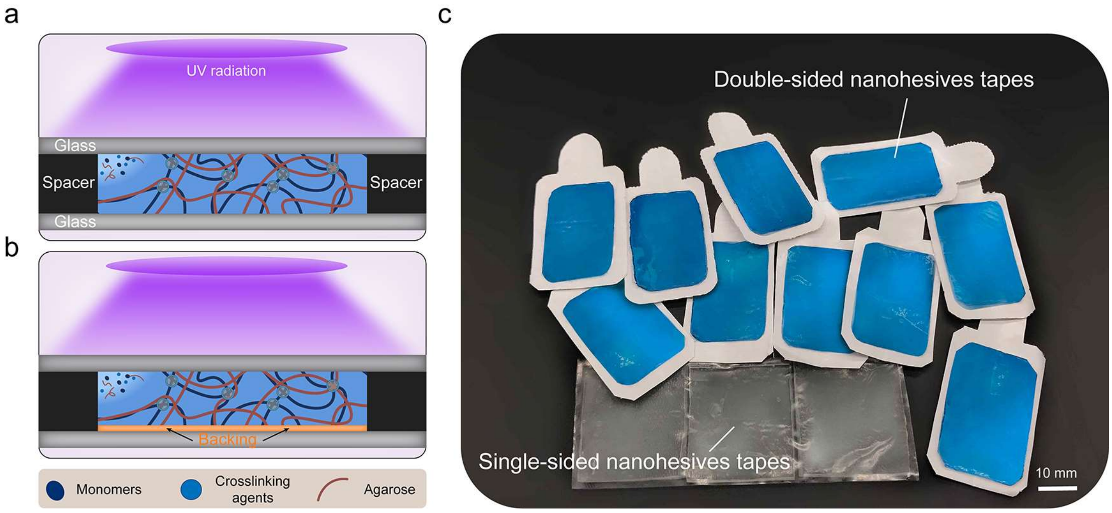

Supplementary Figure 2 The schematic demonstration of the fabrication of nanohesives tapes. The double-sided tapes were fabricated in a template consisting of two glasses (a), while the single-sided tapes were in a template of one glass and one backing (b). The resulted off-the-shelf tapes were demonstrated in (c).

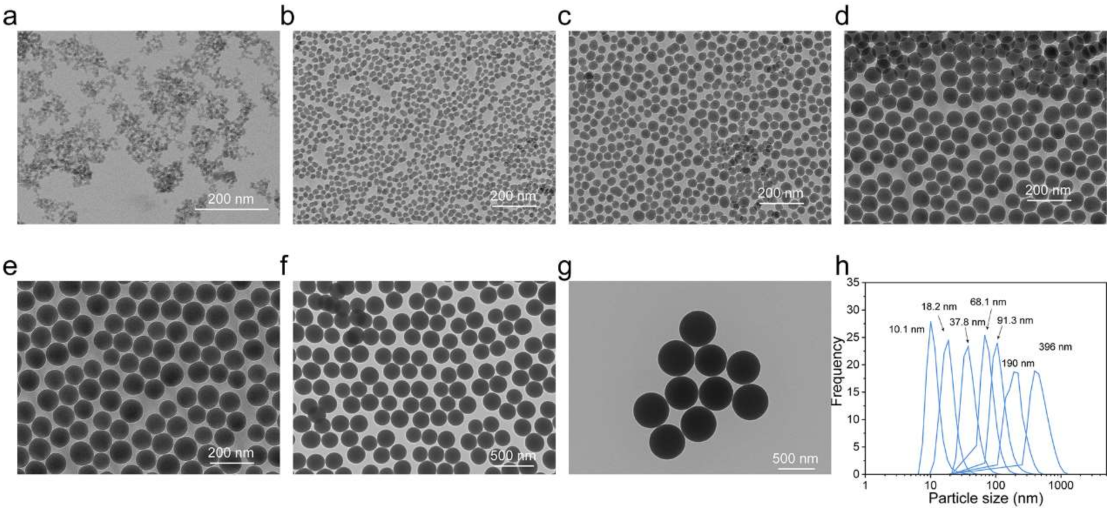

Supplementary Figure 3 a-g, The TEM images of different sizes of ANPs. h, The distribution of hydrodynamic sizes of different ANPs measured by DLS. The experiment was repeated three times independently, and similar results were obtained for TEM observation

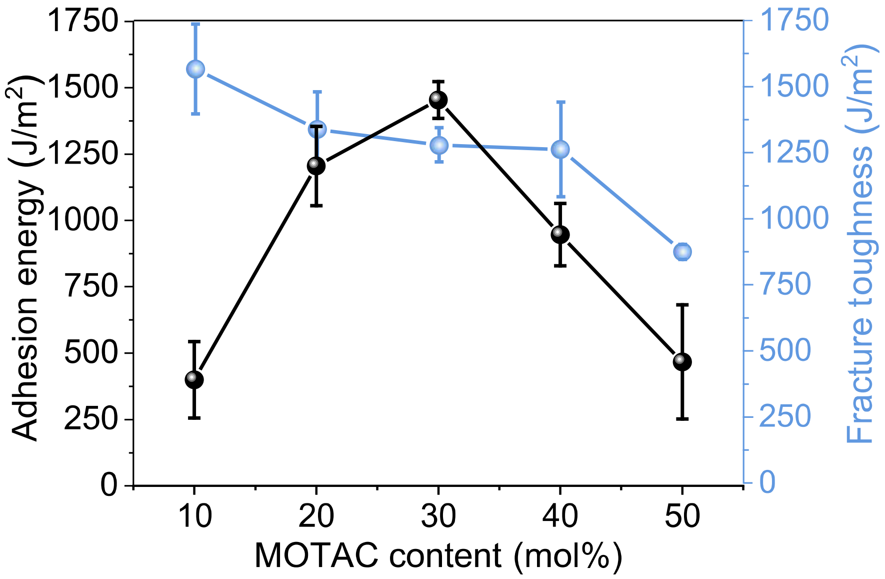

Supplementary Figure 4 The fracture toughness decreases with an increase in MOTAC content, while the adhesive energy exhibits a non-monotonic variation. Values in the panel represent the mean and the standard deviation (n=3, and each represents one independent test).

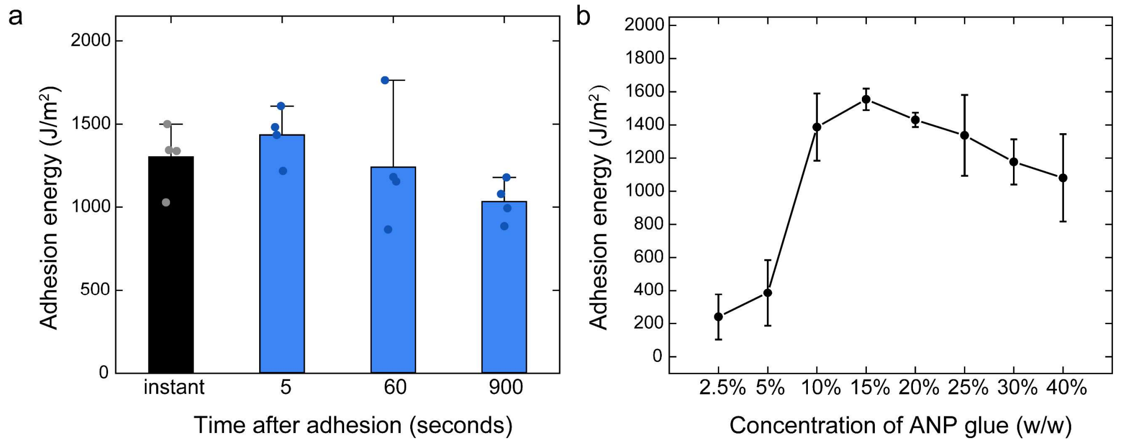

Supplementary Figure 5 a, The relationship between adhesive energy and adhering time. b, The relationship between adhesion energy and the concentration of ANP glue. Values in the panel represent the mean and the standard deviation (s.d.) (*n*=4 independent samples).

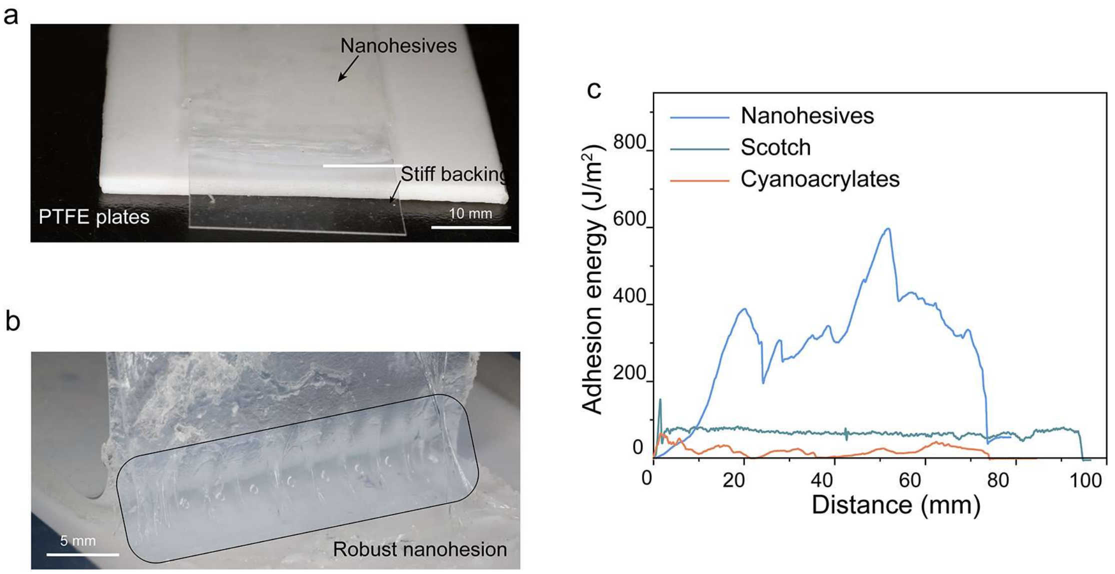

Supplementary Figure 6 Adhesion to PTFE. a, Digital photograph of nanohesives adhered on PTFE. b, Robust adhesion was demonstrated when nanohesives were peeled from PTFE. c, Adhesion energy of peeling tests. The commercial 3M Scotch adhesive tape was directly adhered to PTFE, and the cyanoacrylates substituted ANP glue to adhere dissipative hydrogel on PTFE.

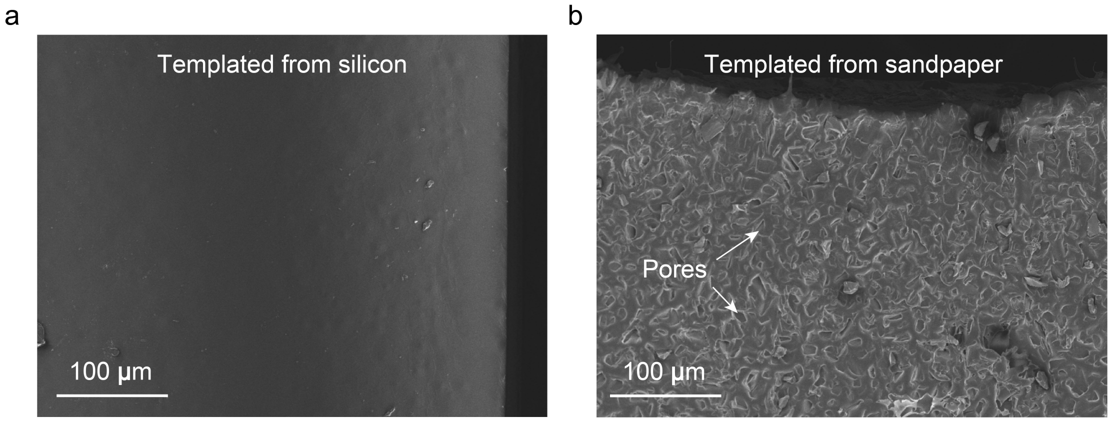

Supplementary Figure 7 SEM images of the surfaces of PLA membranes templated from (a) smooth silicon wafer and (b) 3000 mesh sandpaper. The experiment was repeated three times independently, and similar results were obtained for SEM observation.

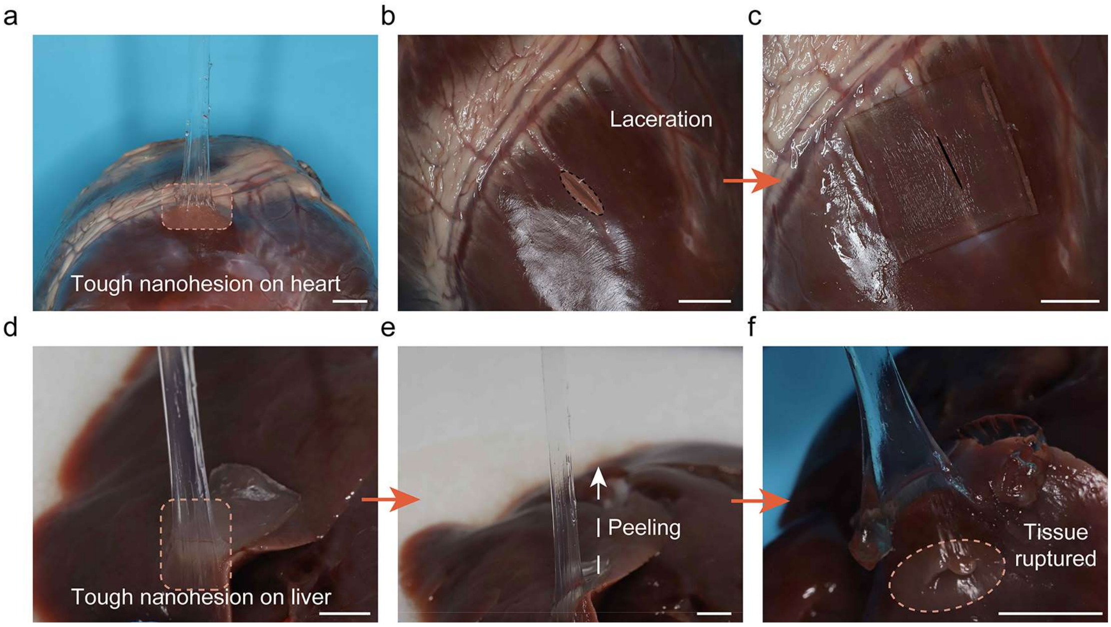

Supplementary Figure 8 a, Nanohesives can robustly adhere to a porcine heart, as well as sealed a lacerated wound on the heart surface seamlessly (b, c). d, The adhesion between nanohesives and the liver was tougher than cohesion of liver tissues, and constant peeling resulted in the rupture of liver tissues before adhesion failure (e, f). Scale bars in pictures represent 10 mm.

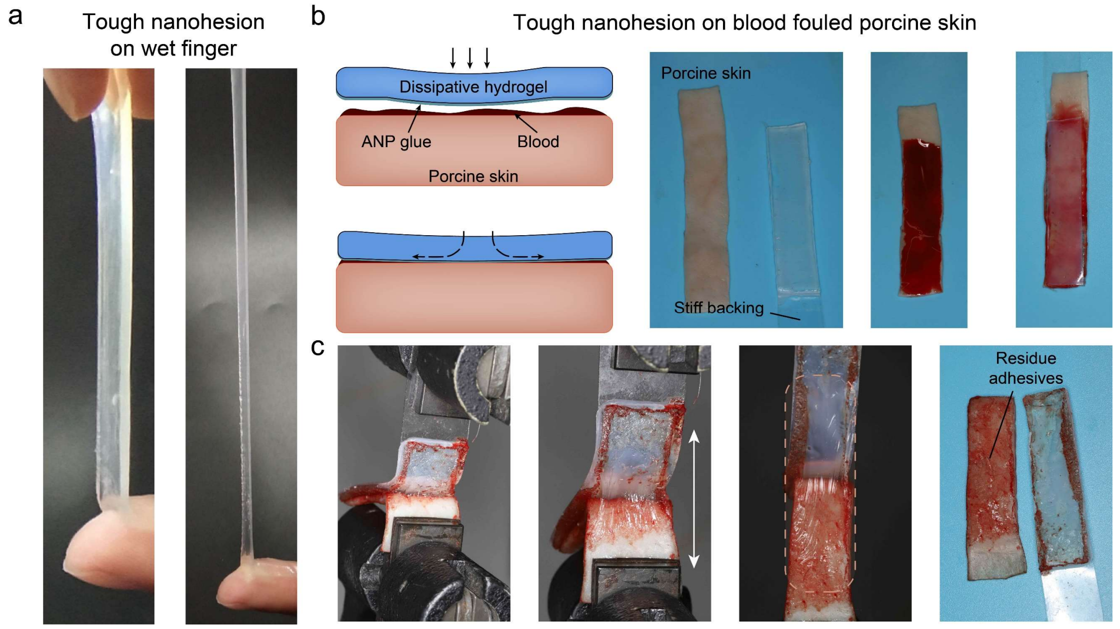

Supplementary Figure 9 a, The tough adhesion of nanohesives on a wet finger of a volunteer, and the volunteer had signed a consent form. b, Blood tolerant adhesion was investigated by covering the whole surface of porcine skin with an excessive amount of whole human blood, before nanohesives were applied. Adhesion was well established after attaching nanohesives onto the skin with gentle press to exclude the blood away from the surface. c, The specimen was stretched by a 180-degree peeling test, and clear fibrillary structures were presented at the crack tip. Most of the blood and adhesive residues remained on the surface of the skin after peeling.

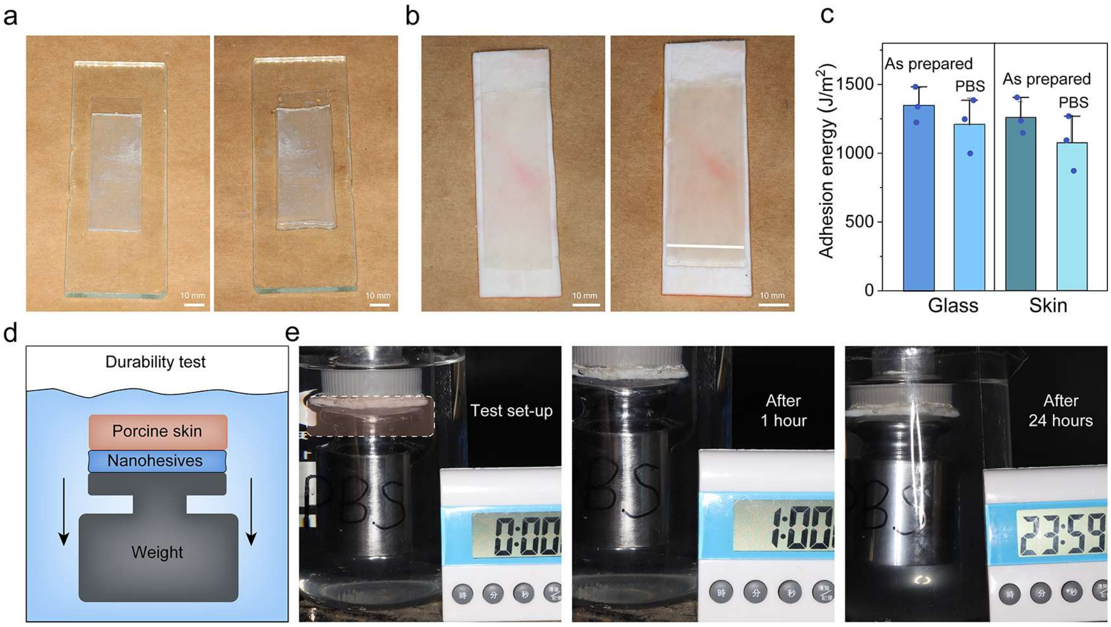

Supplementary Figure 10 Digital photos of stability of nanohesion after being soaked in PBS for 2 days. The specimens were formed by bonding PC membrane and glass (a) or porcine skin (b) together with nanohesives. c, Adhesion energy of specimens that were as-prepared and after being soaked in PBS for 2 days. Values present mean ± standard deviation (s.d.) (n=3 independent samples). d, Stability under stress was tested by bonding a 1 kg steel weight to porcine skin via nanohesives, then hanging up and soaking the set-up in PBS. e, Digital photos of the under-loading adhesive set-up of as-prepared, after being hanged up and soaked for 1 hour, and 24 hours.

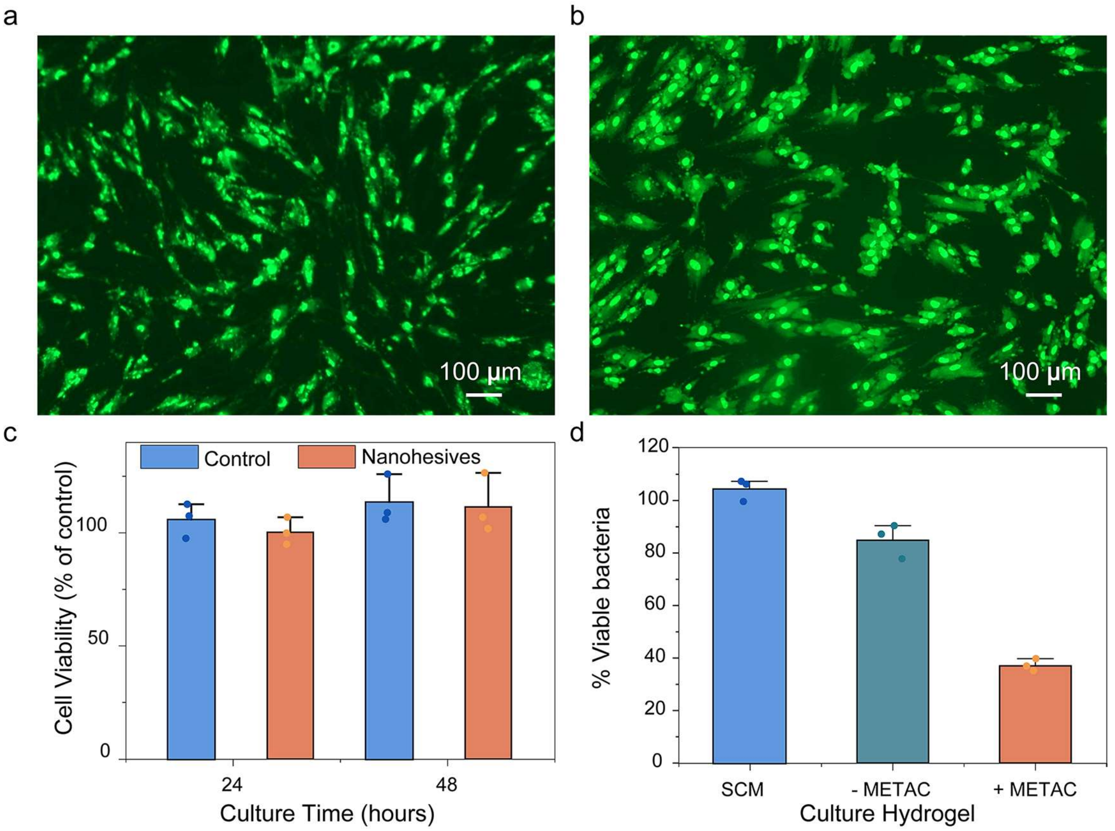

Supplementary Figure 11 a, Fluorescent images of human gingival fibroblasts after 24-hour culture in nanohesives conditioned medium, and (b) DMEM was tested as a control. c, Cell viability was compared between DMEM and nanohesives conditioned medium. d, Viability of Gram-positive bacteria, S. aureus, after 24-hour culture on a normal solid culture medium (SCM), a dissipative hydrogel with and without MOTAC. Scale bars, 100 μm. Values in the panel represent the mean and the standard deviation (s.d.) (*n*=3 independent samples).

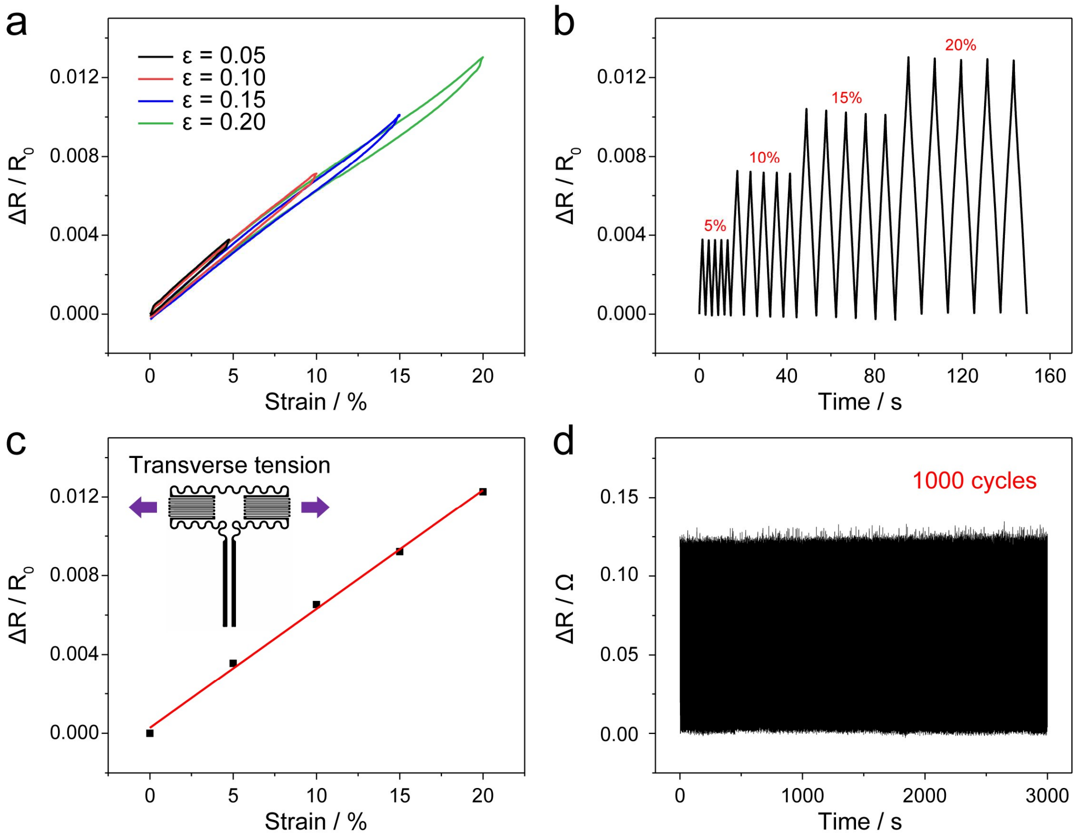

Supplementary Figure 12 a, Cyclic resistance - strain curves, and b, sensitivity, response capability, repeatability test of the strain sensor at ε = 0.05, 0.10, 0.15, 0.20. c, Resistance changes causing by the transverse tension as a function of true strain (obtained by a movie extensometer). The gauge factor is 0.13 in a strain range of 0 - 20 %. d, The lifetime test of the strain sensor under a strain of 5 % in 1000 cycles.

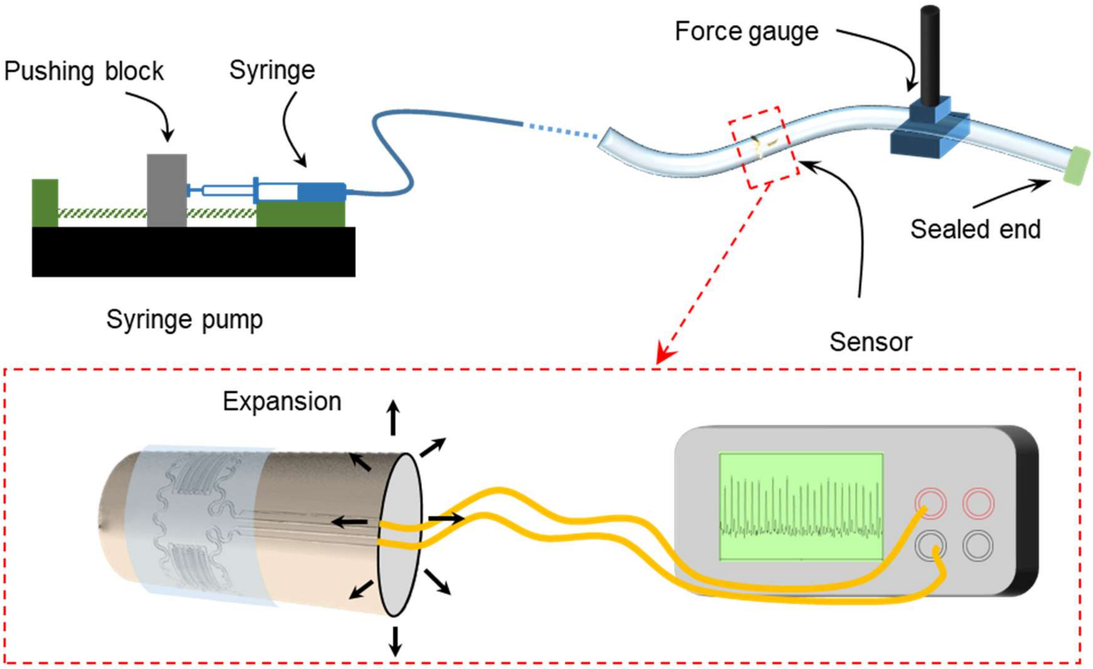

Supplementary Figure 13 The nanohesives-fixed sensor that wrapped around an artificial artery, which was sealed at one end and connected with a syringe pump at the other, was connected to an AVO meter to acquire the pulse signals. The pulsatile behavior was mimicked by the regularly reciprocating pumping from the syringe pump. Simultaneously, the pulse data was also recorded as strain signals from the force gauge as control.

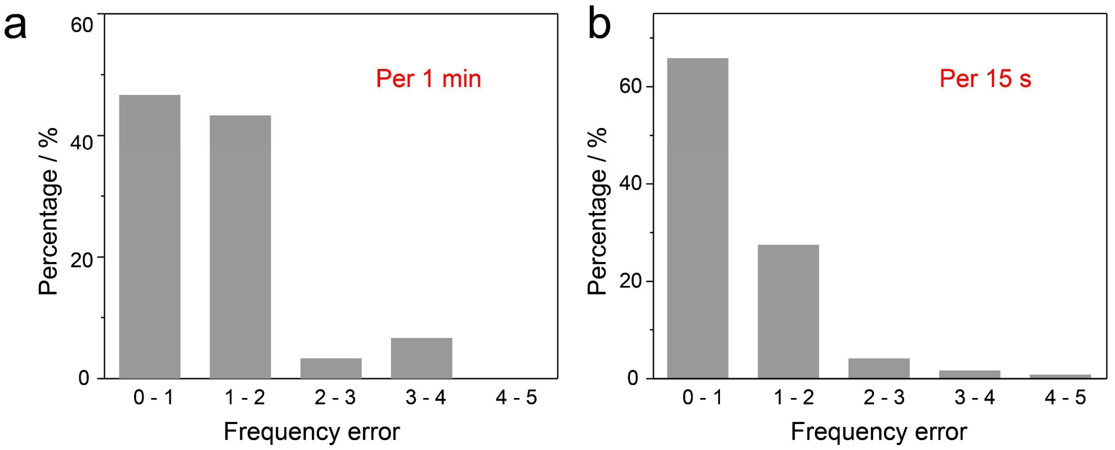

Supplementary Figure 14 In vivo pulse data of the canine femoral artery was simultaneously obtained from the ECG monitor and strain sensor in a 30 min test. The rates were calculated per 1 min (a) and per 15 s (b), and the comparison showed that the difference between NTA fixed sensor detected pulsating rate and standard heart rate was less than 2 b.p.m in more than 90 percent of the whole time.

Supplementary References

[1] Rose, S., et al. Nanoparticle solutions as adhesives for gels and biological tissues. Nature 505, 382-385 (2014).
[2] Masahiro O., et al. Biocompatible nanostructured solid adhesives for biological soft issues. Acta Biomater., 57, 404-413 (2017).
[3] Huang J. X., et al. Combination wound healing using polymer entangled porous nanoadhesive hybrids with robust ROS scavenging and angiogenesis properties. Acta Biomater., 152,171-185 (2022).
[4] Baik, J. S., et al. Colloidal Supraballs of Mesoporous Silica Nanoparticles as Bioresorbable Adhesives for Hydrogels. Chem. Mater. 34, 584-593 (2022).
[5] Kim, J. H., et al. Colloidal mesoporous silica nanoparticles as strong adhesives for hydrogels and biological tissues. ACS Appl. Mater. Interfaces 9, 31469-31477 (2017).
