# Supplementary material for: Designing nanohesives for rapid, universal, and robust hydrogel adhesion
Source: Nat Commun. 2023 Sep 4;14:5378. doi: 10.1038/s41467-023-40753-5 (PMC10477317; doi:10.1038/s41467-023-40753-5)
Supplement: Supplementary file 3 — Description of Additional Supplementary Files [file 41467_2023_40753_MOESM3_ESM.pdf]

Description of Additional Supplementary Files

File Name: Supplementary Movie 1
Description: Formation of adhesion between porcine skins and engineering materials by the nanohesives

File Name: Supplementary Movie 2
Description: Adhesion between nanohesives and Glass via different glue liquids

File Name: Supplementary Movie 3
Description: Rapid formation of strong adhesion between nanohesives and metals

File Name: Supplementary Movie 4
Description: Rapid formation of strong adhesion between nanohesives and ceramics

File Name: Supplementary Movie 5
Description: Rapid formation of strong adhesion between nanohesives and plastics

File Name: Supplementary Movie 6
Description: Rapid formation of strong adhesion between nanohesives and rubbers

File Name: Supplementary Movie 7
Description: Rapid formation of strong adhesion between nanohesives and porcine skin

File Name: Supplementary Movie 8
Description: Formation of adhesion between nanohesives and PBS fouled porcine skin

File Name: Supplementary Movie 9
Description: Formation of adhesion between nanohesives and blood fouled porcine skin

File Name: Supplementary Movie 10
Description: Formation of adhesion between nanohesives and fat side of porcine skin
